# Recent Advances in Minimizing Cadmium Accumulation in Wheat

**DOI:** 10.3390/toxics10040187

**Published:** 2022-04-12

**Authors:** Min Zhou, Zhengguo Li

**Affiliations:** 1 Key Laboratory of Plant Hormones and Development Regulation of Chongqing, School of Life Sciences, Chongqing University, Chongqing 401331, China; 2 Center of Plant Functional Genomics, Institute of Advanced Interdisciplinary Studies, Chongqing University, Chongqing 401331, China

**Keywords:** wheat, Cd accumulation, omics, functional genes, different mitigation strategies

## Abstract

Cadmium (Cd), a toxic heavy metal, affects the yield and quality of crops. Wheat (*Triticum aestivum* L.) can accumulate high Cd content in the grain, which poses a major worldwide hazard to human health. Advances in our understanding of Cd toxicity for plants and humans, different parameters influencing Cd uptake and accumulation, as well as phytoremediation technologies to relieve Cd pollution in wheat have been made very recently. In particular, the molecular mechanisms of wheat under Cd stress have been increasingly recognized. In this review, we focus on the recently described omics and functional genes uncovering Cd stress, as well as different mitigation strategies to reduce Cd toxicity in wheat.

## 1. Introduction

Environmental toxicity from non-essential heavy metals such as cadmium (Cd) is rapidly increasing due to human activities [1,2]. Cd pollution in farmland not only lowers soil quality and decreases crop productivity and quality, but also poses serious threats to the environment and human health, making it one of the most vital environmental concerns globally [3,4,5]. For humans, excessive Cd intake injures the lungs, kidneys, liver, bones, and heart, and its accumulation in human organs causes various diseases such as emphysema, renal and hepatic dysfunction, osteoporosis, and cardiovascular disease [6,7,8,9,10]. For instance, Cd may enhance the liver metabolic burden, and induce abnormal liver function based on the association between blood Cd levels and elevated hematological and hepatic parameters in patients from Cd-exposed and reference groups [8]. For plants, Cd leads to several biochemical and physiological disorders [11]; even when plants are planted in mildly Cd-contaminated soils, Cd can accumulate dramatically in the edible shoot parts, such as the leaves or grains [12,13], which poses a potential hazard to human health [14]. Cereal crops such as wheat, maize, and rice fulfil the staple food requirements of the world. Among the cereals, wheat, one of the most important crops, occupies 17% of cultivated land and serves as the main food source for 60% of the world’s population worldwide [15,16]. Additionally, compared to other cereals, wheat can accumulate more Cd mainly via the roots and translocate it to aerial parts, where it eventually accumulates in the grain [17,18]. Thus, it is important to understand the wheat response to Cd stress and how to manage it for relieving Cd uptake and accumulation, which may help to improve wheat growth, grain yield, and quality. The aim of this review is to summarize the limits for grain Cd content and the human safety threshold, factors that affect Cd uptake and accumulation, toxicity and tolerance mechanisms of Cd stress, recently described omics and functional genes uncovering Cd stress, as well as different mitigation strategies to reduce Cd toxicity in wheat.

## 2. Limits for Grain Cd Content and Human Safety Threshold

Cadmium is one of the most serious heavy metal pollutants, and it poses a potential threat to humans. The situation with Cd pollution has boosted motivation to understand its concentrations in soils and food. Previous studies have reported different ranges of cadmium concentrations in soils and wheat grain worldwide (Table 1). To avoid Cd-related ill-health, the limits for grain Cd content and human Cd concentration or intake have been estimated. The maximum permitted concentration of grain Cd varies in different countries (Table 2). The maximum permitted concentration of grain Cd in Australia is 0.05 mg/kg [19], while in the European Union, it is 0.235 mg/kg [20]. For human Cd intake, the European Food Safety Authority (EFSA) suggested a provisional tolerable weekly intake of 2.5 μg/kg body weight [21]. The Joint FAO/WHO Expert Committee on Food Additives (JECFA) suggested a provisional tolerable monthly intake of 25 μg/kg body weight [22]. The US Agency for Toxic Substances and Disease Registry more recently established a daily minimal risk level of 0.1 μg/kg body weight [23]. Amongst the studies that were reviewed by the EFSA, JECFA, and ATSDR panels, conclusions are different regarding chronic Cd exposure and the amount of body Cd that damages health.

## 3. Factors That Affect Cd Uptake and Accumulation

**Figure 1:**
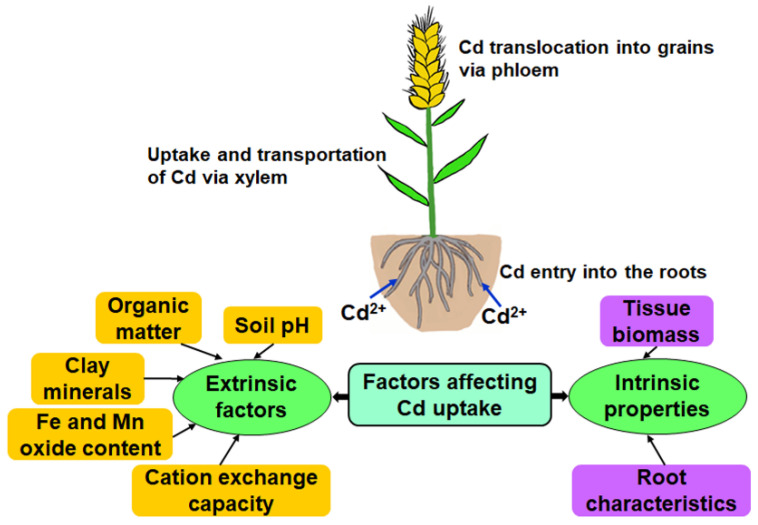
Factors affecting Cd uptake in wheat. Cd first enters the roots from the soil, then after uptake and transport via xylem, it eventually accumulates in the grain via phloem in wheat plants.

Cadmium first enters the roots from the soil, then after uptake and transport via xylem, it eventually accumulates in the grain via phloem in wheat plants (Figure 1). Cadmium availability in soil depends upon various factors (Figure 1), mainly intrinsic properties (such as tissue biomass and root characteristics) [39,40] and extrinsic factors (such as soil factors) [16,41].

It has been reported that Cd content in wheat grain was positively correlated with shoot biomass and root morphological parameters (such as volume, surface area and length) [42]. Liu et al. found that grain Cd concentration correlated positively with the dry weight of the roots and stem, and negatively with the spike length [40]. Recently, Zhang et al. found that root characteristics are important for cadmium tolerance and reduced accumulation in wheat [39]. For instance, longer root length, smaller root diameter, and more numerous root tips were more beneficial for Cd absorption, while thicker roots were able to retain more Cd, thus decreasing root-shoot transport and ameliorating Cd tolerance of the shoots [39].

Cadmium mainly enters wheat plants via the roots [43,44,45]. Thus, soil properties, including soil pH, organic matter, clay minerals, Fe and Mn oxide content, and cation exchange capacity (CEC) can influence Cd uptake by the roots of wheat. The availability of Cd can be increased when soil pH enhances, which expedites the migration of Cd to the grain [46]. Jones and Johnston found a reduction in grain Cd over time in farmyard manure-treated plots, which they explained was due to enhanced Cd retention due to the accumulation of soil organic matter [47]. There was a significant correlation between wheat grain Cd and organic carbon, CEC, and DTPA-extractable Cd concentrations in the soil [16]. When exchangeable Cd is transformed to the more stable fractionation of Fe-Mn oxide bound Cd, this reduces the Cd content in the grains of wheat [48]. Among the above soil properties, soil pH is the most vital factor contributing Cd uptake by wheat plants [46,49,50,51]. Moreover, chloride can mobilize Cd in the soil and enhance its uptake by wheat, particularly by mobilizing inherent soil Cd [52]. In addition, soil Cd bioavailability and Cd uptake by wheat also varies with soil type, atmospheric pollution, and wheat cultivars [46,53,54,55]. For instance, there was a significant but weak negative correlation between soil Cd bioavailability and silt or sand proportion [53]. Gray et al. reported that no strong significant relationship was found between silt content and wheat grain Cd concentration [54].

## 4. Toxicity and Tolerance Mechanism of Cd in Wheat

**Figure 2:**
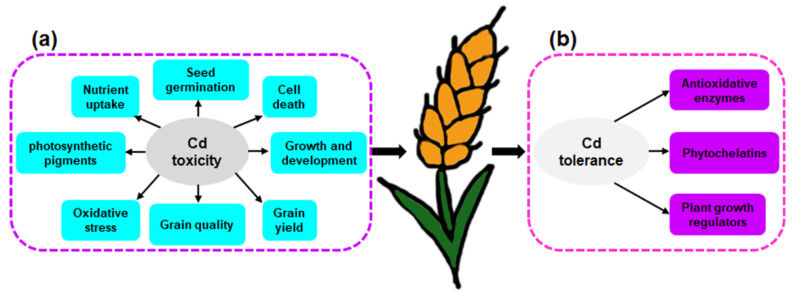
Toxicity and tolerance of Cd for wheat. (**a**) Cd toxicity for wheat. (**b**) Wheat plants evoke several biochemical responses, such as an increase in activity of antioxidative enzymes and overproduction of PCs and PGRs to relieve Cd toxicity.

High Cd concentrations lead to cell death, seriously suppress growth and development, and influence grain yield and quality of wheat plants [56,57,58] (Figure 2a). In wheat, Cd also can affect seed germination, nutrient uptake, photosynthetic pigments, and cause oxidative stress. For example, wheat germination and seedling growth were inhibited by a Cd2+ concentration of 10 μM [59]. Treatment with Cd significantly decreased the concentrations of iron (Fe), magnesium (Mg), calcium (Ca), and potassium (K) in the shoot and leaves of wheat compared to controls [60]. The leaf photosystem II was also damaged in wheat under Cd stress [61]. Moreover, Cd led to oxidative stress as a result of excessive production of reactive oxygen species (ROS) [62].

To deal with Cd toxicity, wheat plants evoke several biochemical responses (Figure 2b) to temporarily alleviate the toxic effects of Cd, such as an increase in the activity of antioxidative enzymes, and overproduction of phytochelatins (PCs) and plant growth regulators (PGRs). Antioxidant enzymes, such as ascorbate peroxidase and superoxide dismutase, and key nonenzymatic antioxidants, such as glutathione and ascorbate, were induced to quench ROS, which are important protective mechanisms to decrease Cd-induced oxidative damage in wheat plants [49,63]. For example, the ascorbate-glutathione (AsA-GSH) cycle ameliorates oxidative stress caused by Cd exposure [64], and glutathione reductase (GR) helps in maintaining the GSH content to ensure proper functioning of the AsA-GSH cycle [65]. Overproduction of PCs also plays an important role in Cd tolerance in wheat [66,67,68]. The PCs are synthesized from GSH and related thiols by glutathione γ-glutamylcysteinyl transferase (γ-ECS), commonly called phytochelatin synthase (PCS), which is activated by a range of heavy metals, including Cd, As, and Pb [69,70]. In addition to PCs, synthesis of plant growth regulators (PGRs), such as salicylic acid and ascorbic acid, were increased to assist wheat tolerate excess Cd [71,72].

## 5. Omics and Functional Genes Uncovering Cd Stress in Wheat

**Figure 3:**
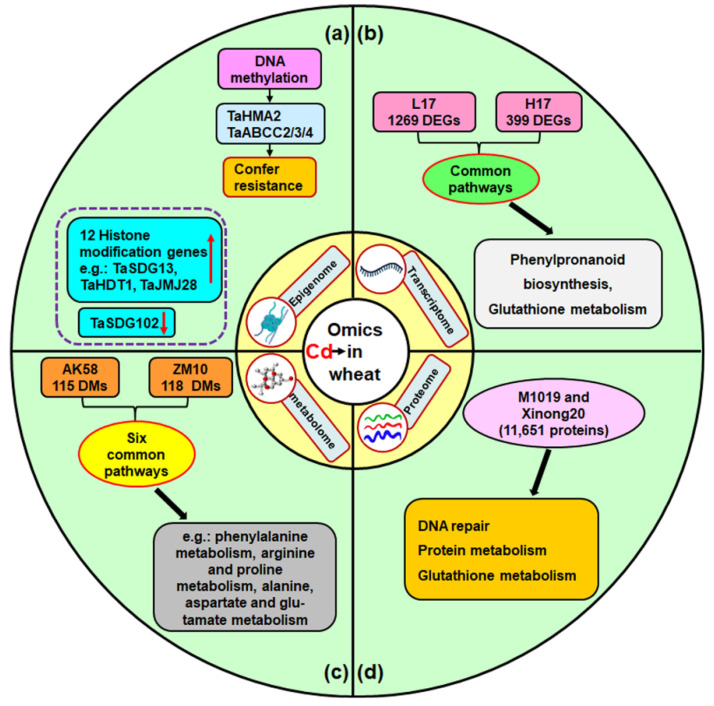
Omics uncovering Cd stress in wheat. Epigenetic response (**a**), transcriptome (**b**), proteome (**c**), and metabolome (**d**) revealing Cd stress in wheat. DEGs, differentially expressed genes; DMs, differential metabolites. Up red arrow represents upregulated, down red arrow represents downregulated.

Omics technologies, such as epigenome, transcriptome, proteome, and metabolome analyses, are now often used in everyday methodology by biological researchers. These technologies have been applied when exploring the response of wheat under various stresses at an extraordinarily detailed molecular level [73]. Recently reported epigenome, transcriptome, proteome, and metabolome analyses of wheat under Cd stress are summarized in the following sections (Figure 3).

### 5.1. Cd Stress and Epigenetic Response

The epigenome is defined as the genome wide composition of heritable or non-heritable chemical modifications of DNA and histone proteins [74]. Accumulating evidence points to the importance of epigenetic modifications that accompany stress induced by environmental contaminants (such as Cd) [75]. These includes several associated processes, such as DNA methylation, histone modifications, chromatin remodeling, and transposable elements [76,77,78]. Epigenetic modifications can affect gene expression, and thus, have vital roles in various biological processes, such as antioxidant defense, metal ion transport, and the response to stress [79,80,81]. It has been reported that DNA methylation regulates the expression of TaHMA2 and ATP-binding Cassette (TaABCC2/3/4) metal detoxification transporters to confer resistance to Cd toxicity in wheat [82] (Figure 3a). For histone modification, Cd treatment induced a 2.2- to 6.4-fold increase in the expression levels of 12 TaHMs (histone modification genes, such as TaSDG13, TaHDT1 and TaJMJ28) in the roots of wheat, but reduced the expression of TaSDG102 (Figure 3a) [83]. Above all, DNA methylation and histone modification plays an important role in wheat plants under Cd stress. However, the responses of other epigenetic modifications (such as RNA m6A modification and chromatin remodeling) to Cd, and whether they are involved in Cd reduction in wheat plants, need to be further investigated.

### 5.2. What about the Transcriptome, Proteome and Cd Stress?

**Figure 4:**
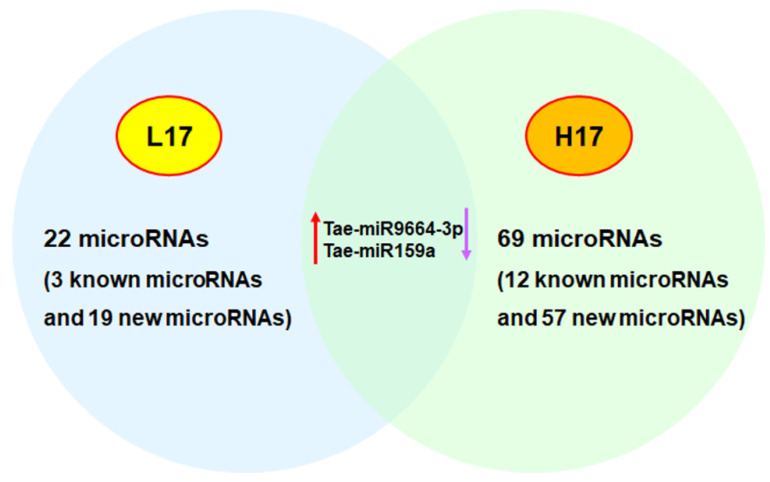
Differentially expressed microRNAs in the roots of two wheat cultivars (L17 and H17) under Cd stress. Tae-miR9664-3p and Tae-miR159a were upregulated in L17Cd (Cd treated) compared to L17CK but downregulated in H17Cd. Up red arrow represents upregulated (Tae-miR9964-3p and Tae-miR159a) in L17Cd compared to L17CK. Down purple arrow represents downregulated in H17Cd compared to H17CK.

Although studies on the proteome and Cd stress in wheat remain in their infancy, it is worth noting the proteome does not always mirror the transcriptome [84]. This finding is vital because that so much of our current understanding of genetic-based diversity comes from transcriptome data. In an early study, Kumari et al. found that mRNA expression for phytochelatin synthase (PCS), glutathione reductase (GR), and ascorbate peroxidase (APX) in roots and leaves were mostly enhanced in two contrasting wheat cultivars under Cd treatment (200 mg/kg soil) using real time PCR, except that PCS was up-regulated significantly in the roots of RAJ4161 (Cd resistant wheat) and down-regulated in PBW343 (Cd sensitive wheat) on day 3 of the Cd treatment [85]. With the development of RNA sequencing and proteomics, these methods have been widely used to investigate the microRNA, mRNA, and protein profiles of plants in response to stress. MicroRNAs (miRNAs) are typically 21 non-coding RNAs that can negatively regulate their target genes via inhibiting or degrading transcripts [86]. Their roles are well established; however, limited knowledge exists about their response to Cd and whether they are involved in Cd reduction in wheat plants. Only a few studies have revealed that Cd stress can induce microRNAs in wheat [87,88,89,90]. Qiu et al. found different microRNA profiles in the root and shoots of wheat seedlings under Cd stress through real-time PCR [89]. Another study by Zhou et al. found a relationship between heavy metal ATPase (HMA) and microRNAs through using a different method in wheat [87]. Zhou et al. found that 22 (3 known microRNAs and 19 new microRNAs) and 69 (12 known microRNAs and 57 new microRNAs) microRNAs were differentially expressed in the roots of two wheat cultivars, low-Cd accumulation (L17) and high-Cd accumulation (H17), respectively, after Cd treatment (Figure 4) [87]. Among them, two special microRNAs (Tae-miR9664-3p and Tae-miR159a) were upregulated in L17Cd (Cd treated), compared to L17CK, but downregulated in H17Cd [87]. They also identified 32 TaHMA genes in wheat. Their results suggested that microRNAs can regulate TaHMAs; however, further verification is required by future research [87]. Various studies have also depicted the mRNA profiles of wheat under Cd stress [91,92,93]. For instance, using RNA sequencing, Zhou et al. identified 1269 differentially expressed genes (DEGs) in L17 (low-Cd accumulation wheat cultivar) after Cd treatment, whereas 399 Cd-induced DEGs were found in H17 (High-Cd accumulation wheat cultivar) (Figure 3b) [91]. Their results also found that DEGs are involved in phenylpropanoid biosynthesis and glutathione metabolism in response to Cd stress in both wheat genotypes. In addition, several studies have depicted the protein profiles of wheat under Cd stress [94,95,96]. For example, Jian et al. found that a total of 11,651 proteins were identified in the roots of two wheat varieties (M1019 and Xinong20) under Cd stress, and the differentially expressed proteins in the two wheat varieties were associated with DNA repair, protein metabolism, and the glutathione metabolism pathway (Figure 3c) [96].

### 5.3. Cd Stress and Metabolome

Metabolome analysis (or metabolomics) has become popular for investigating biosynthetic pathways of interest [97]. Recently, some studies have attempted to investigate the Cd accumulating mechanism in terms of metabolomics profiling for wheat plants [98,99,100]. For instance, Lu et al. investigated the metabolomics profile in the roots of two wheat genotypes, AK58 (Aikang58, a low-Cd-accumulating genotype in grain) and ZM10 (Zhenmai10, a high-Cd-accumulating genotype in grain), under Cd stress. They found that 115 and 118 differential metabolites (DMs) were identified between control and Cd stress for AK58 and ZM10, respectively. Through KEGG analysis, six common potential pathways relating to the antioxidant defense system were identified in the two genotypes, including phenylalanine metabolism, arginine and proline metabolism, alanine, aspartate and glutamate metabolism, isoquinoline alkaloid biosynthesis, arginine biosynthesis, as well as glyoxylate and dicarboxylate metabolism (Figure 3d) [98].

### 5.4. Functional Genes and Cd Stress

With the development of research techniques for gene function, researchers have intensively studied the function of certain genes (Table 3). In an early study, Shim et al. found that heat shock transcription factor A4a (TaHsfA4a) in wheat and rice conferred Cd tolerance by upregulating metallothionein gene expression in planta [101]. Subsequently, to investigate the relationship between the heat shock transcription factor family and Cd stress in wheat, Zhou et al. first identified 78 putative wheat heat shock transcription factor (TaHsf) homologies using wheat genome information [102]. Then, using RNA sequencing and qRT-PCR, they found that TaHsf3 (A1a), TaHsf4 (A2a), TaHsf5 (A2a), TaHsf16 (A3), TaHsf18 (A4a), TaHsf20 (A4a), TaHsf31 (A6b), and TaHsf32 (A6b) were significantly increased in wheat under Cd stress, while levels of TaHsf7 (A2b), TaHsf8 (A2b), TaHsf9 (A2b), TaHsf26 (A5), and TaHsf50 (B4b) were significantly decreased [102]. Heavy metal ATPases (HMAs) have an important role in translocating Cd from plant roots to shoots [87,103]. For example, wheat TaHMA2 can transport Cd^2+^ across membranes [104]. Compared with the wild type, overexpression of TaHMA2 and the TaHMA2 derivative (glutamic substituted for alanine from CCxxE) in Arabidopsis enhanced root length, fresh weight, and increased Cd^2+^ root-to-shoot translocation [104]. Another study found that TaHMA2 on 7B is a candidate gene for grain Cd content in wheat based on the physical location, annotation of candidate gene function, and comparison of homologous genes [24]. Heterologous expression of TaHMA3 genes in yeast revealed no transport activities for Cd, which probably illustrates the low Cd sequestration in wheat roots, and subsequently, the high Cd translocation to wheat shoots. This was evidenced by Zhang et al., since they found that overexpression of the OsHMA3 gene reduced root-to-shoot Cd translocation in wheat by nearly 10-fold and Cd accumulation in wheat grain by 96% [105]. Another study reported that TaHMA3- and TaVP1-encoding proteins related to Cd compartmentalization were significantly upregulated in roots in under Cd stress and was associated with increased Cd tolerance in wheat and reduced Cd translocation to aboveground parts [39]. In addition, overexpression TaCNR5 (cell number regulator 5) in Arabidopsis increased Cd translocation from roots to shoots [106]. Overexpression of durum wheat TdSHN1 conferred Cd tolerance for phytoremediation of heavy metal-contaminated soils by increasing the activities of superoxide dismutase and catalases [107]. Recently, Wei et al. found that overexpression of AetSRG1 (encoding a Fe(II)/2-oxoglutarate-dependent dioxygenase) can decrease Cd accumulation and electrolyte leakage, increase reactive oxygen species production, and promote the synthesis of endogenous salicylic acid by interacting with phenylalanine ammonia lyase (PAL) in wheat [108]. Their results suggest that different genes may be involved in heavy metal detoxification and reactive oxygen species in wheat under Cd stress.

In conclusion, Cd stress-induced epigenetic modifications alters the expression of microRNAs, mRNAs and proteins, and differential metabolites in wheat. More effort is required to investigate the responses of other epigenetic modifications, such as RNA m6A modification and chromatin remodeling to Cd, and whether they are involved in Cd reduction in wheat plants, and to find more functional genes that regulate Cd stress in wheat, so that they can be utilized to improve the phytoremediation ability of wheat via genetic engineering.

## 6. Different Mitigation Strategies to Reduce the Uptake and Accumulation of Cd

**Figure 5:**
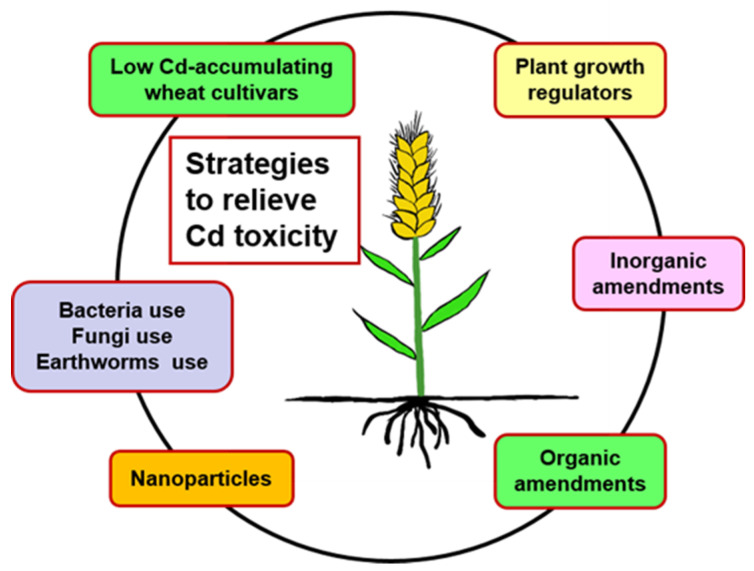
Strategies applied for relieving Cd toxicity in wheat.

Wheat can uptake Cd through its roots and translocate it to the grain, eventually transferring it to humans via the food chain. Therefore, reduction of Cd in wheat is one of the major problems for sustainable agriculture and human health. During the past decades, a variety of alleviation strategies (Figure 5), such as the selection of low Cd-accumulating wheat cultivars, exogenous application of plant growth regulators (PGRs), the use of inorganic amendments, organic amendments, nanoparticles, and biological entities (such as bacteria use, fungi use and earthworms use) have been applied for the management of Cd toxicity in wheat.

### 6.1. Selection of Low Cd-Accumulating Wheat Cultivars

Achieving low Cd-accumulating wheat genotypes through crop variety improvement is one of the most economical and environmentally friendly ways to safely utilize slightly Cd-contaminated soil [39,109]. Previous studies have shown that the Cd content in wheat grain was different among wheat cultivars, even when grown in the same environmental conditions [110,111]. It is also well documented that Cd accumulation in wheat grain varies greatly among wheat genotypes under different environments. For instance, at a high-Cd site, wheat variety JM22 produced significantly lower grain Cd than SX828. At a low-Cd site, significantly lower grain Cd was found in JM22, LX99, and JM262, which could be used as low-Cd cultivars in the study area [112]. Liu et al. selected 24 low-Cd wheat cultivars from 72 wheat cultivars under three different agricultural environments in China [40]. Among the 24 low-Cd wheat cultivars, nine showed stably low-Cd and moderately high micronutrient concentrations in the grain, which are recommended for cultivation in moderately Cd-contaminated farmland. The studies above demonstrated that low Cd-accumulating wheat genotypes could be potentially applied for wheat production in Cd-polluted soils. A breeding strategy might be an option for screening for low Cd accumulation by wheat cultivars to decrease grain Cd content in wheat, which could guarantee food safety. However, the best techniques for breeding low-Cd wheat varieties remain unclear.

### 6.2. Exogenous Application of Plant Growth Regulators

Numerous studies have indicated that the exogenous application of PGRs increases Cd tolerance in wheat. This has been well-reviewed [62]. For example, pretreatment with 500 μM indole-3-acetic acid or 500 μM salicylic acid relieved Cd stress-induced oxidative damage and enhanced Cd tolerance and leaf anatomy by increasing the antioxidant defense in wheat seedlings [113]. Exogenous application of ascorbic acid (AsA) significantly decreased the accumulation of root Cd and increased endogenous ascorbic acid production in wheat after 100 μM Cd treatment, which was associated with NO signaling pathways [114]. A recent study indicated that AsA treatment significantly reduced Cd accumulation in the shoots and roots to relieve Cd toxicity in wheat plants by decreasing MDA accumulation and improving antioxidant defense systems and wheat growth [115]. The studies above mainly investigated the application of PGRs to relieve Cd concentration in roots and shoots of wheat. However, the effect of exogenous application of PGRs on grain Cd accumulation in wheat needs to be explored in future studies.

### 6.3. The Use of Inorganic Amendments

#### 6.3.1. Nitrogen Application

Nitrogen (N) is an essential macronutrient for plant growth, development, and yield [116,117]. However, it is also one of the main limiting nutrients. Numerous studies have reported that application of N fertilizers, such as ammonium nitrate, calcium nitrate, and urea could significantly increase grain Cd accumulation in wheat [118,119,120]. For instance, addition of an ammonium-nitrogen fertilizer at the seedling stage did not affect the grain Cd concentration in Zhoumai (high-Cd accumulator), but dramatically enhanced it in Yunmai 51 (low-Cd accumulator), which indicates that this addition is not suitable for decreasing grain Cd concentration in two common wheat cultivars [118]. Thus, it is important to use a N source that minimizes grain Cd concentrations in wheat.

#### 6.3.2. Phosphorus Application

Phosphorus (P) is one of the most essential macronutrients for crops [121]. Reduction of grain Cd concentration in wheat by using P-containing amendments is well verified. For instance, Ma et al. reported that the grain Cd content of 13 wheat varieties in low-P soil was significantly higher than that in high-P soil [109]. A 10-year field experiment showed that the Cd concentration in the grain of winter wheat (Liangxing 99) was increased by continuous P application (0, 25, 50, 100, 200, and 400 kg P ha^−1^) [122].

#### 6.3.3. Sulfur-Based Fertilizers

Sulfur is an essential macronutrient for plant metabolism, growth, and development [123,124]. It has been reported that sulfur-based fertilizers can alleviate Cd-induced toxicity in wheat. For example, application of elemental sulfur alleviated Cd-induced oxidative stress by regulating ethylene formation, and proline and glutathione metabolism in wheat [125]. Moreover, addition of sodium sulfate reduced Cd concentrations in wheat grain in association with reduced Cd translocation from root and straw to grain, and significantly increased photosynthesis and the growth and grain weight of wheat plants grown in As- and Cd-contaminated soil [126].

#### 6.3.4. Silicon Application

Silicon (Si), the second most abundant element, after oxygen, in the earth crust, is regarded as a quasi-essential element [127]. A protective role of Si for enhancing Cd tolerance is well documented in wheat. For example, Si increased wheat tolerance for Cd toxicity by restricting uptake, accumulation, and translocation of Cd through modulating antioxidative defense mechanisms, including increasing catalase activity, superoxide dismutase activity, and glutathione content [128]. Application of Si increased the soil microbial community (*Acidobacteria* and *Thaumarchaeota*) and bioavailable Si, which significantly reduced soil Cd bioavailability for wheat roots, thus sustaining healthy crop development and food quality [129]. Silicon application decreased Cd accumulation in the roots and shoots by reducing the transpiration rate in a Cd-sensitive cultivar and by enhancing antioxidant activity in a Cd-tolerant cultivar [130]. Furthermore, adding an organosilicon fertilizer and an inorganic silicon fertilizer enhanced Si uptake in the roots and shoots, thus decreasing Cd and Pb accumulation in the shoots, bran, and flour of wheat grown in Cd and Pb co-contaminated soil [131].

#### 6.3.5. Zinc Application

Zinc (Zn) is an essential micronutrient for plants, is an integral part of biochemical function, and plays an important role in maintaining biofilms. Application of Zn can reduce Cd accumulation in the shoots and grain of wheat. For instance, application of 50 μM ZnSO_4_ to H27 (a Cd high-accumulation wheat variety) led to a 17% decrease in Cd concentration in the shoots, while treatment of L979 (a Cd low-accumulation wheat variety) with 100 μM ZnSO_4_ produced a decrease in Cd content. These relieving effects of Zn participated in mechanisms related to root growth, photosynthesis, and antioxidant production for both wheat varieties [132]. Foliar application 0.3% ZnSO_4_ effectively decreased grain Cd content (0.0953 mg pot^−1^) compared with a 30 mg kg^−1^ Cd contaminated control (0.1169 mg pot^−1^) [133]. Foliar application of ZnSO_4_ 7H_2_O in the booting stage effectively decreased grain Cd concentration and minimized Cd-induced loss in grain yield for wheat (cv ARRI-2011) grown in three levels of soil Cd (0, 2.5, and 5.0 mg kg^−1^) [134]. Soil (99 kg ZnSO_4_ 7H_2_O ha^−1^) and foliar (0.36 kg ZnSO_4_ 7H_2_O ha^−1^) Zn applications can effectively reduce Cd in the grain of wheat [135].

### 6.4. The Use of Organic Amendments

#### 6.4.1. Composts and Manures

Composts and manures, derived from many different sources, have commonly been applied in many bioremediation experiments to decrease heavy metal bioavailability in contaminated soils [136]. For example, farmyard manure application decreased grain Cd concentration compared to the application of urine and digestate [52]. Long-term organic fertilization with composted manure or green waste compost reduced the Cd concentrations in the shoots and grain of winter wheat [137]. Field management with farmyard manure plus limestone decreased grain Cd concentration and increased plant yield in wheat grown in a Cd-contaminated field [138].

#### 6.4.2. Biochar

Biochar, a byproduct of the thermal decomposition of biomass under limited or non-oxygen conditions at high temperatures, has the potential to control the bioavailability and transformation of heavy metals such as cadmium in soil [139]. For example, rice husk biochar application significantly decreased Cd concentrations in wheat root, shoot, and grain [140]. Rice straw biochar also reduced Cd content in roots, shoots and grain, and increased antioxidant enzyme activities, and morphological and physiological parameters when wheat was grown under combined Cd and drought stress [141]. In addition, Majeed et al. compared the effects of rich straw biochar, maize stalk biochar, farmyard manure, and pressmud applied at a rate of 1% *w*/*w* on wheat grown in Cd-spiked soil (6.0 mg/kg), and found that the application of maize stalk biochar was more efficient for reducing Cd content in the leaves and grain of wheat compared to other organic amendments [142].

### 6.5. The Use of Nanoparticles

Nanotechnology has been progressively used in agriculture to improve crops and it can largely deal with the shortcomings of genetic and traditional agronomic biofortification [143]. It is well documented that various nanoparticles can improve Cd tolerance in wheat through different methods, such as seed priming, foliar application, and soil application. For example, seed priming with zinc oxide (ZnO) and iron (Fe) nanoparticles significantly decreased Cd concentrations in roots, shoots, and grain, reduced oxidative stress, and increased the biomass (plant height, spike length, dry weights of shoots, roots, spikes, and grain) and nutrients (Zn and Fe concentrations, respectively) in wheat grown under Cd stress [144]. For foliar application, it has been reported that zinc oxide nanoparticles decreased grain Cd concentrations and oxidative stress, and increased leaf superoxide dismutase and peroxidase activities, in wheat grown under simultaneous Cd and water deficient stress [145]. Hussain et al. [146] also found that foliar application of ZnO nanoparticles, Fe nanoparticles, and Si nanoparticles lessened Cd intake, which benefited wheat growth, yield, and nutrient uptake by plants grown in Cd-contaminated soil under real field conditions. In addition, soil amended with zinc oxide nanoparticles significantly decreased Cd accumulation in tissues and grains by decreasing soil-bioavailable Cd and its accumulation by roots [147]. Treatment of soil with 100 mg/kg of copper nanoparticles synthesized by a copper-resistant bacterium *Shigella flexneri* SNT22 enhanced plant length, shoot dry weight, nitrogen and phosphorus content, and reduced acropetal Cd translocation [148]. Other research reported that both foliar spray and soil application of Si nanoparticles reduced the Cd content of shoots, roots, and grains of wheat grown under Cd toxicity [149]. The studies above suggest that nanotechnology-based biofortification is one effective method to relieve Cd toxicity in wheat. However, a rigorous evaluation of the safety of the nanomaterials needs to be carried out to improve its application for alleviating Cd pollution in field-scale programs.

### 6.6. The Use of Biological Entities

#### 6.6.1. The Use of Bacteria

The use of biological entities, such as bacteria, have been reported in mitigating wheat Cd toxicity by several studies. Bacteria not only decreases Cd concentration in the roots and shoots through several factors, they also reduce grain Cd accumulation in wheat cultivated in Cd-polluted conditions. For example, wheat inoculated with *Azospirillum brasilense* strain Az39 showed lower Cd levels in their roots compared to non-inoculated wheat plants [150]. *Ralstonia eutropha* Q2-8 can relieve Cd toxicity in wheat plant seedlings and decrease above-ground tissue Cd uptake by enhancing the efficiency of the root energy metabolism and cell wall biosynthesis under Cd (5 mM) + As (10 mM) stress [151]. Cd-resistant bacteria strain WRS8 isolated from wheat rhizosphere soil decreased wheat tissue Cd accumulation by enhancing root surface Cd adsorption and reducing wheat root Cd uptake and transport-related gene expression (such as LCT1 and HMA2) [152]. Moreover, application of the metal(loid)-resistant bacteria *Ralstonia eutropha* Q2-8 and *Exiguobacterium aurantiacum* Q3-11 enhanced soil pH and the abundance of genes possibly involved in metal(loid) unavailability, resulting in decreased root and grain Cd accumulation in wheat grown in Cd- and As-polluted soils [153].

#### 6.6.2. The Use of Fungi

Arbuscular mycorrhizal fungi are broadly distributed plant symbiotic fungi in natural and agricultural soils, and they play a vital role in promoting the phytoremediation of heavy metal-polluted soils [154,155]. In wheat, it has been reported that arbuscular mycorrhizal fungus (*Glomus veruciforme*)-inoculated wheat plants under Cd stress had increased amounts of chlorophyll, total soluble sugars, and total proteins in the shoots and roots compared to non-arbuscular mycorrhizal fungus-inoculated plants [156]. Shahabivand et al. found that another arbuscular mycorrhizal fungus, *Glomus mosseae*, significantly increased shoot length, shoot dry weight, chlorophyll content, and reduced root Cd content in wheat under Cd stress compared with non-inoculated plants [157]. They also found that *Piriformospora indica* (a root enophytic fungus) inoculation produced greater readings for growth parameters and chlorophyll content, and decreased shoot Cd accumulation, than *Glomus mosseae* inoculation of wheat. Furthermore, Baghaie et al. [158] reported that addition of soil-indigenous arbuscular mycorrhizal fungi to soil can reduce Cd uptake by the grains of bread wheat.

#### 6.6.3. The Use of Earthworms

Earthworms are generally distributed on earth and occupy about 60–90% of the belowground soil biomass [159]. Several recent studies have demonstrated that the combination of earthworms and plants or microorganisms is promising for Cd-contaminated soil remediation [160,161]. In wheat plants, Lai et al. [162] reported that the coexistence of earthworms with wheat enhanced plant biomass and decreased Cd content in wheat roots and shoots. Present studies have mainly investigated the application of earthworms for Cd-contaminated soil remediation and the mitigation of Cd concentration in the roots and shoots of wheat. However, the effect of earthworm use on grain Cd accumulation in wheat needs to be explored in future studies.

### 6.7. The Use of Combined Strategies

Several studies have focused on the effect of combined strategies on alleviating Cd toxicity in wheat. For example, an interactive effect of NO and H_2_S can considerably ameliorate wheat resistance to Cd toxicity by decreasing oxidative stress and uptake of Cd in wheat plants, as well as by increasing the antioxidative defense system and uptake of some essential mineral nutrients [163]. The combined application of Si and NO increased the efficacy of treatment for Cd toxicity in wheat seedlings compared to sole treatments by up-regulating the antioxidant defense system (particularly AsA-GSH cycle) [164]. Aside from combining two inorganic amendments, some research has concentrated on combining two organic amendments, or an inorganic amendment with an organic amendment. For instance, co-composted farm manure and biochar decreased grain Cd concentration, increased chlorophyll content, growth and yield, and minimized the oxidative stress in the leaves of wheat under drought stress [165]. Wheat receiving the combined application of an inorganic (citric acid chelate) and an organic (*Bacillus* sp.) treatment showed decreased Cd content in the grain [166]. In addition, Han et al. reported that *Enterobacter bugandensis* TJ6 combined with sheep manure reduced Cd content (75%) in wheat grain by improving rhizosphere soil urease activity, NH_4_^+^/NO_3_^−^ ratio, and pH [167]. The studies above suggest that it is effective to apply combined strategies to alleviate Cd toxicity in wheat. Further studies are required to better understand the mechanisms behind the different additives for the mitigation of Cd accumulation in wheat, especially at the transcriptomic and proteomic levels.

## 7. Summary and Perspectives

Cadmium concentration has been increasing substantially in the environment to severely affect the growth, grain yield, and quality of wheat [168,169]. Its presence has been regarded as a serious threat to agriculture and human health. Excess Cd leads to oxidative stress and genotoxicity in wheat plants. Thus, they evoke the antioxidant defense system and regulate ion homeostasis and signaling molecules to deal with Cd toxicity. The minimization of Cd pollution in wheat is urgently needed around the world. At present, several strategies have been successfully applied for minimizing Cd toxicity at the experimental stage for wheat. These strategies mainly include low Cd-accumulating wheat cultivars, exogenous application of PGRs, the use of inorganic and organic amendments, nanoparticles, bacteria, fungi, and earthworms. Cultivation of low Cd-accumulating wheat varieties along with other suitable strategies might be an effective way to produce safe and high-quality grain with a low Cd content. There are still challenges for the practical techniques for breeding low-Cd wheat cultivars and the field application of a variety of strategies for minimizing Cd pollution in wheat plants. In addition, omics such as epigenomics, transcriptomics, proteomics and metabonomics have been applied to investigate Cd toxicity at the molecular level in wheat. However, the key genes involved in wheat under Cd stress are poorly reported. More work is needed to address these issues.

In summary, although great progress has been achieved, substantial efforts are still required to find practical techniques for breeding low Cd wheat cultivars, apply various Cd minimization strategies in the field, as well as address the regulatory networks of wheat under Cd stress. It is also urgent that scientists research other epigenetic responses to Cd stress in wheat, such as RNA m6A modification and chromatin remodeling, and explore how to utilize functional genes to improve the phytoremediation ability of wheat via genetic engineering.

## Figures and Tables

**Table 1:** Cd content in soil and wheat worldwide.

Cd in Soil (mg/kg)	Cd in Wheat Grain (mg/kg)	Area	References
0.57	0.083–0.126	Soda Springs, United States	[24]
0.38	0.048–0.145	Zhejiang province, China	[25]
2.06	0.10–0.25	Henan province, China	[26]
0.099–1.007	0.006–0.179	Kunshan, China	[27]
Not reported	0.9317	Lahore, Pakistan	[28]
0.21	0.015–0.083	Sao Gotardo, Brazil	[29]
3.20	0.01–0.03	Qom, Iran	[30]
Not reported	0.003–0.07	Sydney, Australia	[31]

**Table 2:** Maximum permitted concentration of grain Cd in individual countries.

Country	Maximum Permitted Concentration of Grain Cd (mg/kg DW)	References
Australia	0.05	[19]
Canada	0.20	[32,33]
China	0.10	[27]
Croatia	0.20	[34]
European Union	0.235	[20]
Iran	0.20	[30]
Italy	0.20	[35,36]
Japan	0.20	[37]
New Zealand	0.10	[20]
Pakistan	0.10	[28]
Serbian	0.20	[38]
United States	0.20	[33]

**Table 3:** Functional genes in wheat under Cd stress.

Gene	Function	References
TaHsfA4a	Confer Cd tolerance by upregulating metallothionein gene expression	[101]
TaHMA2	Transport Cd^2+^ across membranes	[104]
OsHMA3	Reduce root-to-shoot Cd translocation in wheat and Cd accumulation in wheat grain	[105]
TaHMA3 and TaVP1	Increase Cd tolerance in wheat and reduce Cd translocation to aboveground parts	[39]
TaCNR5	Increase Cd translocation from roots to shoots	[106]
TdSHN1	Confer Cd tolerances by increasing activities of superoxide dismutase and catalases	[107]
AetSRG1	Decrease Cd accumulation and electrolyte leakage, increase reactive oxygen species production and promote the synthesis of endogenous salicylic acid through interacting with phenylalanine ammonia lyase	[108]

## Data Availability

Not applicable.
